# Commentary: A new uPAR-targeting fluorescent probe for optical guided intracranial surgery in resection of a meningioma-a case report

**DOI:** 10.1007/s00701-022-05410-8

**Published:** 2022-11-11

**Authors:** Eric Suero-Molina, Michael Müther

**Affiliations:** Department of Neurosurgery, University Hospital of Münster, Albert-Schweitzer-Campus 1, A1, 48149 Münster, Germany

Dear Editor,

With great interest, we have read the recent publication by Skjøth-Rasmussen and co-workers on a novel fluorescent probe for intraoperative detection of brain tumors, which appears to overcome some of the limitations of 5-ALA, a traditionally used intra-operative fluorophore [1].

They report on a patient with suspected high-grade glioma operated on using the novel probe with the tumor turning out to be a meningioma. They report unexpected strong, tumor-linked fluorescence observed with the ORBEYE® exoscope device and corroborate tumor selectivity with several positively fluorescing tumor biopsies.

According to the authors, the probe, a urokinase-type plasminogen activator receptor (uPAR) targeting ligand, is available in two versions, one for PET imaging and one for fluorescent-guided surgery (when conjugated with ICG) and—in the future—possibly even for treatment when used as a uPAR-targeting radionuclide. Thus, the probe might be the ideal theranostic agent for tumors expressing uPAR.

While this report is promising, some of the possible confounders involved in developing an intravenous fluorescent probe are not discussed. Firstly, when injected, the concentration of intravenous probes will be high in plasma and lead to time-dependent fluorescence in tissue before being extravasated into the tumor. Extravasation into the tumor will depend on blood–brain barrier disruption since there are no known transporters of uPAR targeting transporters nor channels at the blood-brain barrier. Much of the accumulation in the meningioma may well only be passive in nature because a meningioma has no blood-brain barrier excluding uPAR targeting moieties, as would also be the case for a high-grade glioma in its enhancing areas. This would have little to do with true tumor cell affinity. It is unclear (due to the molecular size of the fluorescing construct) whether the normal human blood-brain barrier will let uPAR-targeting ligands pass into the brain, which may be a problem with low-grade gliomas, for example. How can the BBB barrier’s pseudoaffinity due to intratumoral breakdown be differentiated from true tumor affinity?

**Fig. 1:**
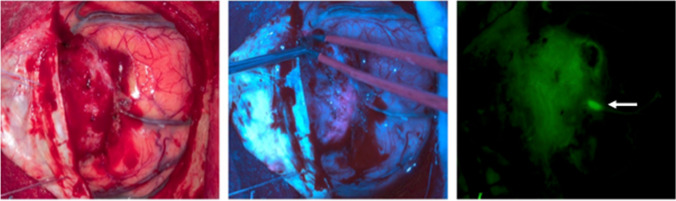
Unspecific fluorescence at the surgical margins and in an apparently thrombosed vein (arrow) by Skjøth-Rasmussen et al.

On the other hand, circulating complex will still lead to brain fluorescence in a time-dependent manner. It will be extravasated when tissue is injured, leading to unspecific fluorescence at the surgical margins. This can be observed in Fig. 2 of the author’s report (Fig. 1 in this letter), where the agent is visible at the right margin of the brain, in the lower margin in tissue, and in an apparently thrombosed vein (arrow).

Besides the time dependency of the contrast ratio between brain and tumor, corroborating fluorescence using biopsies is a highly biased process. Others have defined several possible biases and confounders which need to be respected during validation studies of fluorescent agents [2–4] based on biopsies. To overcome such biases, studies certainly require a certain degree of complexity to be accepted as approval studies by FDA and EMA for intra-operative imaging agents [5]. This standard is listed in the EQUATOR network database (https://www.equator-network.org/reporting-guidelines/intraoperative-fluorescence-diagnosis-in-the-brain-a-systematic-review-and-suggestions-for-future-standards-on-reporting-diagnostic-accuracy-and-clinical-utility/).

While the authors are to be congratulated on their work for the future development of their probe for fluorescence-guided resections, they should be mindful of some of the recent developments, also for regulatory approval, for the design and reporting of their future studies for their highly interesting approach to fruitfully fulfill regulatory requirements and make this method available to patients.

Sincerely,

Eric Suero-Molina

Michael Müther
